# Supplementary material for: Are carbon emissions trading and green financial instruments synergistic? -Comprehensive quantitative research based on content analysis
Source: PLoS One. 2024 Mar 7;19(3):e0298601. doi: 10.1371/journal.pone.0298601 (PMC10919601; doi:10.1371/journal.pone.0298601)
Supplement: S1 Data — (ZIP) [file pone.0298601.s001.zip › Data/地级市Z_F工作报告.zip!/╣π╬≈/╖└│╟╕█╩╨/└┤╫╘╣1⁄2╓┌║┼í╛┬φ┐╦╩2╛▌═°í┐_2019.docx]

各位代表：

　　现在，我代表市人民政府向大会作政府工作报告，请予审议，并请市政协各位委员和列席的同志提出意见。

　　一、2018年工作回顾

　　2018年，在自治区党委、政府和市委的坚强领导下，在市人大、市政协的监督支持下，我们紧紧依靠全市各族人民，全面贯彻党的十九大精神，以习近平新时代中国特色社会主义思想为指导，坚持稳中求进工作总基调，贯彻新发展理念，以深化供给侧结构性改革为主线，持续打好三大攻坚战，以“四大活动”为牵引，以“三个年活动”为抓手，统筹稳增长、促改革、调结构、惠民生、防风险，全市经济社会发展取得新成绩，较好地完成市六届人大四次会议确定的年度主要任务。

　　经济运行呈现新态势。GDP增长7.4%，其中第一产业增加值增长6%，第二产业增加值增长7.2%，第三产业增加值增长8.2%。规上工业增加值增长8.5%，规模以上万元工业增加值能耗同比下降2.7%。社会消费品零售总额增长8.8%，居民消费价格指数涨幅3.4%。财政收入增长11.1%，排全区第四；全口径税收收入增长20.8%，排全区第二；非税占比25.8%，排全区第一，经济运行质量效益持续提升。

　　转型升级获得新成效。三次产业结构优化，第三产业增加值占比提高5.6个百分点，旅游消费贡献率61.4%，排名全区第一，“互联网+”等新业态蓬勃发展，电商零售销售额增长29.9%。盛隆等一批企业技改成效显著，大海粮油等4家企业成为国家级两化融合管理体系贯标试点企业。农业产业结构加快调整，农林牧渔业总产值增长6.3%。

　　重大项目取得新突破。推进自治区统筹项目和市“六个一百”项目建设，分别完成投资204.7亿元和265.6亿元。柳钢防城港钢铁基地、生态铝基地实现实质性开工，核电华龙一号3号机组穹顶顺利吊装。中海油LNG项目竣工试产。防东铁路正式进场施工。G219峒中至东兴公路获得交通部重大项目专项资金支持。南宁经上思至防城港、上思至峒中、东兴至凭祥、沿海高速钦州至防城港段等公路纳入广西高速公路网规划。

　　工业企业效益实现新提升。规上工业企业利润总额增长12.6%。精炼铜、精制食用植物油、钢材等工业产品实现两位数增长。盛隆上榜中国企业500强、名列广西民企榜首，金川等5家企业入围广西企业100强，红沙核电获自治区主席质量奖，澳加粮油、中电获评广西首批智能工厂示范企业，华润水泥（上思）获评广西首批绿色制造示范工厂，经开区工业投资总量在全区114个园区中排名第一。

　　城市品牌建设彰显新魅力。防城港边境旅游试验区获批设立，跨境自驾游常态开通，东兴口岸年通关人次突破1200万，居我国边境陆路口岸之首。公共文化服务示范区终期评审排西部第三。生态环境质量保持广西前列，空气质量优良天数比率95.3%。成功举办中国—东盟国际马拉松、北部湾首届开海节、北部湾全国桥牌赛等重大文体活动，城市知名度美誉度大幅提升。

　　民生福祉取得新进步。城镇居民和农村居民人均可支配收入分别增长7%、9.3%，排全区前列。城镇登记失业率1.28%，为全区最低。成为全国首批社保标准化建设先行城市。海湾新区10所学校全部开工建设。多项卫生计生服务指标排全区第一。全年4859人脱贫，16个贫困村脱贫摘帽。

　　各位代表！2018年我市先后举行庆祝自治区成立60周年座谈会、建市25周年暨建港50周年主题情景报告会等一系列活动。孙春兰副总理率中央代表团二分团到我市慰问，对我市取得的成就给予高度评价，极大地鼓舞了全市广大干部群众。建市25年来，在党中央和自治区的坚强领导下，全市上下众志成城，奋发图强，港城大地发生翻天覆地的变化，从昔日小渔村华丽转身为一个美丽生态海湾城市、现代化新兴港口工业城市，从原来的开放末梢走向沿海沿边开放前沿，从温饱不足走向小康和富裕，全市各族人民无不感到自豪与骄傲，对防城港的美好未来满怀激情、充满信心！

　　过去一年，我们主要做了以下工作：

　　（一）着力筑牢基础稳增长。贯彻中央“六稳”部署，扎实推进经济运行平稳较快增长。抓好重大项目稳定有效投资。以“项目提速年”为抓手，以“六个一百”项目为重点，开展产业大招商活动，全年招商到位资金639亿元，同比增长11.4%，引进50亿元以上项目总投资额排全区第二。举行10次项目集中开竣工活动，197个项目实现开竣工，计划总投资783.8亿元。钢铁基地、生态铝基地、核电二期顺利推进。建成渔澫港区501#泊位等一批港口码头项目。钦防高速改扩建、企茅一级路、第五作业区进港航道等一批交通项目加快建设。防城港支线机场等一批重大项目前期工作稳步推进。力促工业经济企稳向好。以“产业提升年”为抓手，加快打造广西冶金产业二次创业核心区，推动盛隆冶金等企业通过技改做大做强。新增15家工业企业上规入统。改革城镇供水投融资模式，推进增量配备电网建设，工业用水、用电价格有效降低，冷链集装箱铁路运价下调43%，累计帮助实体经济企业降低成本达30亿元。规上工业企业主营业务利润率5.5%，改变以往产值与增加值相背离的状况，实现同步增长。积极支持民营经济发展。落实国家和自治区支持民营经济发展政策，全年新引进民间投资项目117个，总投资386.6亿元，同比增长45.1%。推动政府性融资担保公司有效运营，扩大民营和小微企业融资业务。开展政银企对接，设立信贷引导资金，累计发放“惠企贷”5.53亿元。发展产业投资基金，总规模达26亿元。加大财政金融支持力度。停征、取消3项行政事业性收费，全年减轻企业负担超1亿元。出台政府投资引导基金管理暂行办法，促进财政资金以投代补服务实体经济。完善转贷资金运作机制，有效纾解企业融资难融资贵融资慢问题。

　　（二）着力培育壮大新动能。坚持从传统产业挖掘新动能，从转型升级中嫁接新动能，从新产业、新技术、新业态中培育新动能。推动工业高质量发展。树立“工业树”理念，全力打造“产业林”，培育钢、铜、铝冶炼等产业，推动企业产品差异化发展。做强核电、火电等能源产业，做大粮食、油料精深加工食品制造业，打造铜基新材料产业基地。发展现代服务业。加快边境旅游试验区规划建设，完善边境旅游基础设施，创新中越跨境旅游“六联合”模式。东兴跨境电子商务发展指数进入全国县域20强，位居西部第一。加快发展冷链经济，率先在国内实现东盟海产品冷链公铁海联运，防城港车站累计发送冷链产品3.2万吨，同比增长24%。稳步推进保税物流中心（B型）建设。夯实科技创新基础。高新区云朗科技园一期投入运营，沙潭江生态科技园启动区加快建设。科研投入占GDP比重排全区第三位。高新技术企业数量达26家，增长18%，创历年新高。新增院士工作站2家，建成自治区级农业科技园区和科技企业孵化器各3家。每万人发明专利拥有量4.06件，增长10.3%。盛隆公司参与制定《热轧带肋钢筋》国家标准获批实施。推动数字防城港建设，大数据产业加快发展。

　　（三）着力推进全方位开放。主动融入西部陆海新通道建设。落实自治区 “南向、北联、东融、西合”部署，开展开放合作北联行动，先后与自贡、攀枝花、宜宾、泸州等缔结友好城市，成功举办成都南向通道投资项目推介会。顺利入选港口型、边境口岸型国家物流枢纽布局承载城市。夯实基础促进开放。建成中越峒中—横模口岸桥等一批口岸设施，扎实做好北仑河二桥、云约江作业区泊位开放筹备。成功入选广西境外旅客购物离境退税政策首批试点城市，与泰国黎逸市结为国际友城。扩大对外贸易，全年港口货物吞吐量增长0.9%，集装箱31万标箱，增长16%，外贸进出口总额完成721亿元，排名全区第三。

　　（四）着力深化重点领域改革。深化管理体制改革。推进国资国企整合重组、市管开发区管理体制和城市管理工作机制改革，企沙镇获批广西首批经济发达镇行政体制改革试点。出台政府性工程变更办法，改革财评制度。持续抓好“放管服”改革。深化“一事通办”改革，“最多跑一次”事项占全部事项的99.6%。推行“四个一”行政审批新机制，实现 “354560”改革目标。开展“减证便民”专项行动，最大限度利企便民。全面公布权力清单和责任清单，在国内率先实施34证合一，启动全国首个设区市“证照分离”改革试点。统筹做好其他改革。健全土地资源盘活及统征统迁制度，推行“净地出让+资金共管+阳光招拍挂”模式，全年盘活800多公顷存量土地。沿边金融改革创造3个全国第一、多个全区第一。东兴试验区入围“改革开放40年地方改革创新案例”名单。

　　（五）着力推进城乡统筹发展。加快城市规划建设。率先在全区启动国土空间规划，编制新一轮珍珠湾片区概念性规划、河西片区控规等一批规划，严把规划控制关，坚守城市天际线。打通江山大道等一批断头路，建成海湾绿道35公里，完成一批城市主干道白改黑。加快园博园、伏波大道等项目建设，防城江东岸片区初具规模，城南片区与中心片区融为一体，高新区与海湾新区连成一片，港产城融合发展新格局加快形成。商品房销售面积同比增长60.5%，人气商气日益兴旺。扎实推进乡村振兴战略。围绕 “六大工程”“两大行动”部署，重点推进东兴桂产业、上思皇袍山森林生态文化旅游等一批核心示范区建设，建成国家级产业园1个、自治区级示范区5个、县级示范区9个、乡级示范园45个。新增7个农产品“广西名牌”。规划建设企沙、白龙双墩、东兴天鹅湾三大渔港经济区，京岛海洋渔业、港口红树林海洋渔业两个核心示范区分别获自治区四星、三星认定。40个自然村屯获评广西“绿色村屯”，53个行政村获评自治区级生态村。农村人居环境整治三年行动取得阶段性成效。京族哈节入选首届“中国农民丰收节”，京族海洋特色小镇、金花茶特色小镇列入广西首批特色小镇培育名单。

　　（六）着力打好三大攻坚战。打好扶贫攻坚战。投入财政专项扶贫资金3.14亿元，特色产业扶贫覆盖率达80%以上，易地扶贫搬迁进度加快，预脱贫的16个贫困村和51个脱贫村的集体收入均达到3万元以上。打赢污染防治攻坚战。抓好中央环保督察“回头看”反馈问题整改。大气、水、土壤攻坚取得明显成效，空气优良率连续四年全区第一，集中式饮用水源地水质达标率100%，农用地土壤详查采样工作基本完成。切实防范化解金融风险。认真履行属地金融风险处置监管责任，做到早预警、早处置，堵住风险源头。打击非法集资、金融犯罪、网络传销等活动。积极化解政府债务，筹措偿债资金33.8亿元，累计置换存量债务105.9亿元，每年减少利息3亿元，政府债务率大幅下降。

　　（七）着力发展民生社会事业。舍得拿出最好的资源保障改善民生，舍得拿出最好的地给老百姓安置，舍得拿出最多的钱为民办实事。全年民生重点领域支出100.8亿元，占一般公共预算支出近八成。加强精神文明建设。抓好市本级创建自治区文明城市以及东兴市创建全国文明城市工作。港口区山新村等2个村被评为全国文明村镇，市检察院等4家单位被评为全国文明单位，实现了市（县）直单位争创全国文明单位零突破。打造首批市级社会主义核心价值观示范点45个。发展文体事业。国家公共文化服务体系示范区通过终期验收，“国门文化大院”“文缘号海上图书流动船”等创建活动被中宣部、文旅部列为全国示范推广项目。成功举办海上龙舟节、中国—东盟城市网球赛、国际海钓赛等文体活动。补齐教育卫生事业短板。投入约9.7亿元建设海湾新区10所学校。四个县（市、区）均通过义务教育均衡发展国家级评估认定。高考成绩取得历史性突破，600分以上考生增长213%。北部湾高中在全国公开引进名校长，学校管理进一步强化。开展国家第四批城市公立医院改革，公立医疗机构参与医联体、医共体、专科联盟建设率100%。推进市第一人民医院迁建、市妇幼保健院迁建等一批卫生攻坚项目建设。上思县获评全区计划生育优质服务先进单位。做好社会保障工作。基本医保、基本养老保险参保完成率分别排名全区第一、第二名。城市、农村低保保障标准分别增长21.6%、19.4%。边民补助提标扩面，补助标准从每人每月130元提高到167元，惠及边民16.5万人。全力办好民生实事。全年为民办十件实事项目计划总投资24亿元，人大代表票决的2018年民生实事项目基本完成，妥善解决多年未落实的海珍路小商品市场安全隐患整治、港口区“98户”群众宅基地安置等一批社会关注的民生问题，取得良好社会效果，赢得了广大人民群众的高度赞扬。

　　一年来，我们坚持以全面从严治党统领政府各项工作，加强党风廉政建设，抓好巡视发现问题整改。加强制度建设，结合“大学习、大调研、大策划、大落实”活动，推行“学懂用”、项目“1+6”、绩效“1+4”等制度，推动各项工作提速提质提效。坚持依法行政，落实法律顾问制度，改进行政复议、行政应诉工作。健全重大行政决策机制，加强审计监督和行政监察，推动决策、执行、管理、服务和结果公开。依法接受人大及其常委会的监督，自觉接受人民政协的民主监督，人大代表建议和政协提案办结率100％。

　　国防动员、人民防空和边海防、民兵预备役、双拥、救灾减灾、安全生产、应急管理等工作成效显著。“扫黑除恶”专项斗争、反走私、禁毒等社会治理扎实推进，人民群众安全感不断提升。加强民族宗教事务管理，外事侨务、海关、边检、消防救援、海事、市场管理、信访、档案保密、统计、供销、粮食、通信、地震、水库移民、农机、社会科学、地方志、工会、共青团、妇女儿童、老龄、残疾人、红十字、机关事务等工作都取得新进展。全市边疆稳定，民族团结，人民安居乐业！

　　各位代表！过去一年，我们求真务实，勇于创新，解决了不少难题，办成了许多好事实事，防城港发展迎来了新的机遇，焕发了新的生机。奋斗饱含艰辛，成绩来之不易。这是自治区党委、政府和市委正确领导的结果，是市人大、市政协监督支持的结果，是全市人民团结奋斗的结果。在此，我代表市人民政府，向全市各族人民，向人大代表、政协委员，向我市驻军、武警官兵、消防救援指战员和中直、区直单位，向各民主党派、工商联、无党派人士、人民团体，向关心支持防城港发展建设的港澳台同胞、海外侨胞及海内外朋友们，表示崇高的敬意和衷心的感谢！

　　在肯定成绩的同时，也要清醒看到发展中存在的困难和问题：经济总量小，产业支撑能力不足，新旧动能转换较慢，投资和消费增长放缓；实体经济面临困难较多，民营经济发展活力不足；县域经济比较薄弱，扶贫、教育、卫生、城建等方面短板还较突出；营商环境亟待优化，政府工作仍需改进等。我们要迎难而上，勇于担当，用新理念新方法认真加以克服解决。

　　二、2019年经济社会发展总体要求和主要目标

　　今年是新中国成立70周年，是全面建成小康社会关键之年。党中央、自治区党委政府和市委提出了一系列的新部署新要求，我们要结合实际，创造性地贯彻落实好。今年政府工作的总体要求是：以习近平新时代中国特色社会主义思想为指导，深入贯彻落实党的十九大和十九届二中、三中全会精神，统筹推进“五位一体”总体布局和协调推进“四个全面”战略布局，深入落实“三大定位”新使命和“五个扎实”新要求，坚持稳中求进工作总基调，坚持新发展理念，坚持以供给侧结构性改革为主线，坚持市场化改革、扩大高水平开放，统筹推进稳增长、促改革、调结构、惠民生、防风险工作，进一步稳就业、稳金融、稳外贸、稳外资、稳投资、稳预期，着力推动产业高质量发展，着力扩大市场需求，着力促进县域协调发展，着力抓好脱贫攻坚和民生建设，以海强市，以边旺市，以山富市，加快构建海边山现代化经济体系，为全面建成小康社会收官打下决定性基础，以优异成绩迎接中华人民共和国70周年华诞。

　　今年经济社会发展主要预期目标是：地区生产总值增长7.5%左右，财政收入增长6%，规模以上工业增加值增长8.5%，固定资产投资增长10%，社会消费品零售总额增长10%，外贸进出口总额增长5%，城镇居民人均可支配收入增长7%，农村居民人均可支配收入增长9%，城镇登记失业率控制在4%以内，居民消费价格涨幅控制在3%左右，节能减排降碳控制在自治区下达目标内。

　　提出上述目标，体现了中央、自治区决策部署和我市实际有机结合的要求，努力走出具有我市特色的发展路子；体现了稳中求进的要求，努力保持经济运行在合理区间；体现了推进高质量发展的要求，努力转变发展方式、优化经济结构和转换增长动力；体现了以人民为中心的要求，努力让人民过上更加美好生活。完成今年目标任务，我们拥有良好基础和支撑条件：在工业上，我们有一批重大项目支撑。盛隆技改、杭氧空分装置和益海嘉里发酵豆粕等项目年内投产，柳钢冷轧项目等一批减产企业复产、增产。预计全年规上工业增加值增长8.5%左右，第二产业增长8%左右。在服务业上，我们有许多新的增长点。边境旅游试验区加快建设，马拉松、开海节等文旅品牌影响力不断扩大，冷链经济迅速发展，交通客货运周转量、批发零售和住宿餐饮等指标呈增长态势，预计服务业增加值增长8%左右。在农业上，我们有一批发展势头良好的特色产业。农林牧渔业大有潜力可挖。粮食生产稳定，海产品、水果、肉类等农产品产量持续增长。预计今年全市农林牧渔业总产值增长5%、第一产业增长4.8%以上。在投资上，我们将迎来新的投资高峰期。近年来上马了一批重大项目，集中开工了一批补短板项目，有相当部分结转今年建设。钢铁、中铝、盛隆技改、防东铁路将大幅拉动有效投资，预计全年固投增长10%左右。

　　当前经济形势与政策环境对我市总体有利，我们要坚定信心，真抓实干，着重从以下方面努力：必须认真贯彻落实上级部署。按照“三大定位”“五个扎实”和“建设壮美广西”的新要求，结合市情和阶段特征，对标找差、对表落实，精心谋划好各项工作，推动习总书记重要指示精神和中央、自治区各项决策部署在我市落地见效。必须深入挖掘海边山优势。聚集发展要素资源，在构建海边山现代化经济体系上持续探索、创造新的优势，以更大作为开创我市经济社会发展新局面。必须大力培育经济发展新动能。抓住国家积极财政政策加力提效、稳健货币政策松紧适度的机遇，加紧在投资、消费、县域经济等方面培育新增长点。在产业升级、基础建设和乡村振兴等领域谋划实施一批工程项目，促进新动能茁壮成长。必须加快补齐民生短板。按照全面建成小康社会要求，围绕城乡教育、卫生、扶贫、交通、饮水、基础设施配套等短板弱项，抓住工作关键，重点突破，务求实效。

　　三、2019年主要工作安排

　　今年经济社会发展任务艰巨繁重，我们要突出重点，扎实做好以下工作：

　　（一）增强创新支撑能力，推动工业高质量发展

　　推进工业转型升级。按“强龙头、补链条、聚集群”思路，不断突破制约产业结构优化升级的关键环节。抓好重大产业项目。重点推进钢铁基地、生态铝基地、铜基新材料产业基地建设，续建核电二期、澳加粮油、大海粮油技改等项目。加快推进盛隆技改、科元ABS装置等项目竣工投产，启动金川铜冶炼二期和冶金上下游配套等项目，开工经开区集中供热、中储能电解液及储能新材料产业园等项目。争取国家核准核电三期项目。优化传统工业产业。对接自治区冶金二次创业“一核三带九基地”布局，加快对钢铁、有色、能源、粮油等传统产业进行技术改造，提升产业链水平。推进信息化和工业化深度融合，争做广西新型工业化发展引领者。促进产业园区提档升级。推进园区产业加快集聚，经开区要重点发展钢铁、有色、能源、新材料、先进制造等产业，高新区要加快发展信息技术、生物医药、新材料、先进制造等新兴产业，培育壮大现代服务业。探索实施“一区多园”“大园带小园”等模式，开展园区产业链合作。规划防城港制造城建设。聚焦智能制造、建筑工程装备、电子信息、新能源汽车等领域，在经开区、高新区等区域谋划建设防城港制造城，力争明年入围自治区制造城建设项目。

　　筑牢科技创新支撑。实施科技创新支撑产业高质量发展行动，促进科技与产业融合发展。强化企业创新主体地位。加快培育高新技术企业，支持中储能暖泉、小藻科技等一批科技型中小企业加快发展。依托龙头企业建立产业研究院，促进科技成果在一线转化。深化“产学研”合作，推进盛隆冶金与研究所合作研发石墨烯基耐腐蚀钢材产业化发展，增强企业自主创新能力。夯实科技创新平台。加快云朗科技园、沙潭江生态科技产业园等园中园建设。利用IT小镇门户等载体，引进一批高成长科技型企业。建好工程技术研究中心、院士专家工作站、产业技术创新战略联盟等创新平台，支持企业开展新型学徒制试点。发展数字经济。聚焦中国—东盟数据大通道、西南多式联运物流大数据、跨境电商等重点领域，推进产业数字化，建设一批智能化工厂，培育一批“四新”企业。积极抢占5G时代先机，助力数字防城港发展。

　　发展壮大民营经济。深入实施非公有制强优企业培育计划，鼓励“个转企”“小升规”，有效增加“四上”企业数量。完善联系服务非公企业工作制度，密切政商关系，维护民营企业合法权益。积极作为，靠前服务，全力为民营企业解决准入门槛高、融资难、经营成本高、发展活力不足、历史遗留问题多等问题，让港城大地成为民企投资置业新热土！

　　（二）增加重大项目有效投资，夯实经济发展基础

　　抓好重大基础设施项目。公路方面，争取开工南宁经上思至防城港高速公路、G219峒中至东兴公路等项目；续建企茅一级路、沙企一级路改扩建等项目，推进国门大道项目建设；确保钦防高速改扩建项目竣工通车。铁路方面，加快建设防东铁路、钢铁基地铁路专用线；争取开工南防铁路复线工程；抓好茅岭至皇城坳、潭油作业区等铁路支线项目前期工作。港航方面，续建渔澫港区513—516号泊位等项目，开工401号、赤沙作业区1、2号三个20万吨级泊位和潭油航道一期等项目，抓好30万吨级矿石码头及配套航道等项目前期工作。口岸方面，加快建设北仑河二桥、峒中等口岸基础设施，确保口岸扩大开放顺利通过国家验收。推动华立集团大型边境综合产业项目落地建设。能源方面，续建防城南山风电场工程等项目，开工中电防城港电厂余热综合利用、门崖岭风电场工程等项目，推动高新区增量配电网建设。民航方面，推进防城港支线机场、通用机场项目规划建设。

　　抓好城市建设项目。加快编制市域国土空间规划，为新一轮城市建设打下良好基础。高水平建设海湾新区，抓好大平坡路等一批城市路网建设，基本完成主干道白改黑工程。尽快开工市民中心、城市第二水源地供水管网等项目。加大市政消火栓建设力度，完善城市消防设施。推进城南片区建设，建成“堤路园”，确保广西园林园艺博览会如期举行。改造防城老城区，有效疏解拥堵和低品位开发问题。完善渔澫片区，持续提升城市功能品质。加快经开区、高新区、江山旅游区与主城区一体化建设，推动港产城深度融合发展。建设绿色阳台、园林、公园等一批城市景观，打通西湾环海绿道，提高环海环岛景观亮化水平。以公园城市理念，重塑城市肌理，打造壮美新港城！

　　抓好重大社会事业项目。统筹整合相关渠道资金，加大社会民生补短板力度。教育方面，续建海湾新区10所新建学校、理工职业学校二期等项目，开工实验高中改扩建、防城中学学生宿舍楼、特殊教育学校康复楼等项目。医疗卫生方面，加快推进市第一人民医院迁建、港口分院升级改造、市妇幼保健院业务用房等建设项目，创建市三级甲等综合医院。文体方面，推进市体育中心等一批项目的前期工作，加快建设东兴文化馆、图书馆等项目。

　　完善项目工作机制。优化“1+6”项目工作制度，健全厅级领导联系重大项目制度，继续抓好项目集中开竣工，营造谋项目、引项目、抓项目、成项目良好氛围。改革项目资金支持方式，集中资金优先保障重大项目。强化首问负责制、限时办结制、服务承诺制等制度管理，推行“一线工作法”，解决好项目建设“最先一公里”和“最后一公里”的问题。

　　（三）推进乡村振兴战略，开创农业农村发展新局面

　　深入实施乡村振兴工程。抓好“六大工程”和“两大行动”，发展现代特色农业，推进“一村一品”建设。坚持绿色发展，保护农村生态环境。建好“新时代讲习所”，发展海边山乡村特色文化。加强农村基础设施建设，增加乡村公共服务供给。深入推进农村人居环境整治三年行动计划，提升农村人居环境质量。健全农村社会保障体系，夯实脱贫致富支撑。完善农村基本经营制度，提升小农户组织化程度。打造境外农作物原料生产基地等开放合作平台，构建边境特色乡村振兴示范带。培养更多有一技之长的乡土人才，造就一批懂经营、善管理的致富能手。

　　发展现代特色农业。深入开展“10+3”行动，抓好19万亩粮食生产功能区、45万亩糖料蔗生产保护区划定。推动特色农业示范区增点扩面，建成自治区级核心示范区2个，县乡村三级示范区（园）118个。加快玉桂八角、油茶、沃柑、肉牛等产品产业化发展，推动金花茶产业振兴。发展休闲农业，推进京岛海洋特色小镇建设，打造沿海沿边线、南山生态线等乡村旅游线路。强化农业科技推广，抓好农业物联网试验示范。

　　发展海洋渔业。加快建设企沙、白龙双墩、东兴天鹅湾三大渔港经济区。搞好珍珠湾海洋牧场建设，建成陆基工厂化循环水养殖基地5万平方米、深海抗风浪网箱养殖水体20万立方米。支持集约化海水健康养殖，发展生态渔业和设施渔业，拓展远洋渔业，抓好水产品精深加工。

　　发展农产品加工业。推进江平海洋产业园、冲榄工业园和企沙渔港经济区等园区水产加工业建设，规划建设防城互市贸易商品落地加工园区。支持主产区依托县域形成农产品加工产业集群，推动就近加工增值、全产业链发展。构建冷链经济体系，加快桂海国际农产品冷链物流中心、广西物资集团冷链产业园等项目建设，建成一批示范性冷库、交易中心、加工基地和电商产业园。

　　推动糖业发展壮大。推动糖料蔗规模化生产，年内完成1万亩糖料蔗“双高”基地建设任务。拓展甘蔗综合利用新领域，推进蔗渣生物质发电，提高朗姆酒、木糖醇等高附加值产品生产水平，抓好蔗叶、滤泥等综合利用，促进牛羊养殖和环保生态肥料生产。推动上思农垦朗姆风情甘蔗循环经济核心示范区建设，争取创建自治区五星级示范区，加快打造朗姆小镇。

　　完善农村基础设施。加快“四好农村路”建设，未来两年要完成60公里县乡道联网路和133公里县乡道安全隐患治理。推进乡乡通二级路建设，在有条件的乡村建设一批集中供水工程。大规模推进高标准农田建设。抓好123个水利建设项目前期工作，实施新一轮农村电网升级改造，推动天然气进乡入村。

　　建设美丽防城港幸福乡村。推进乡村风貌提升三年行动，开展“环境秀美”“生活甜美”“乡村和美”三个专项活动。以垃圾污水治理、厕所革命、村容村貌提升为重点，建设一批环境整治精品示范村庄。编制行政村实用性规划，整治村庄公共空间，建设一批村级公共服务中心，培育文明乡风、良好家风和淳朴民风。

　　（四）聚焦重点精准发力，坚决打好脱贫攻坚战

　　狠抓中央巡视组反馈意见整改。贯彻落实中央第二巡视组专项巡视情况反馈意见，推动脱贫攻坚工作落细落地。坚持把脱贫攻坚作为重大政治任务和头等大事来抓，不能因我市深度贫困人口相对少而忽视，不能有麻痹大意、懈怠侥幸的心理。对标对表中央巡视反馈和自治区整改意见，聚焦脱贫攻坚基础工作薄弱、执行政策不精准、资金使用进度慢、措施落实不到位、帮扶工作不扎实等突出问题，压实主体责任，高质量打赢脱贫攻坚战，确保年内4800名贫困人口脱贫，9个贫困村脱贫摘帽。

　　努力消除深度极度贫困。消除深度极度贫困是打赢脱贫攻坚战的重中之重，必须强化特殊扶持。加大对上思米强村、防城那巴村等深度贫困村的扶持力度，把握边境地区扶贫重点，精准推进0－3公里边境地区5个贫困村2607名贫困人口脱贫，在财政资金、用地指标、就业等方面给予倾斜。做好贫困地区义务教育、基本医疗、住房安全“三个保障”。抓好贫困户危房改造，按建设成本对极度贫困户危房改造全额补助。确保有劳动能力的极度贫困户每户安排1个公益性岗位。

　　夯实稳定脱贫基础。抓好产业扶贫，推广“贷牛还牛”、联建联养、扶贫车间等新模式，对有发展能力的贫困户实现产业扶贫全覆盖。发展村级集体经济，通过龙头企业、农民合作社、创业致富带头人等经营主体带动特色产业发展，确保每个贫困村集体经济年收入4万元以上。搞好易地扶贫搬迁，完成建档立卡贫困人口搬迁入住，强化易地扶贫搬迁后续管理，帮助搬迁群众稳定就业。加强贫困地区路、水、电、通信基础设施建设，基本实现20户以上自然屯通硬化路、10－20户自然屯通砂石路。

　　（五）主动融入西部陆海新通道建设，形成全方位开放新格局

　　积极参与西部陆海新通道建设。这是我市落实“三大定位”新使命、提升“海陆双通道”“南向门户城”新优势的重要抓手。建设港口型国家物流枢纽。加快建设一批港航工程，改善进出口航道条件，提升港口陆海联运、水水中转和国际中转等功能，为中南西南地区及东盟国家提供货物集散、转口贸易、保税监管等物流服务和其他增值服务。建设边境口岸型国家物流枢纽。做好防城港、东兴、峒中等口岸扩大开放验收工作，恢复开通江山港等边地贸口岸。建设东兴口岸交通物流枢纽，完善东湾物流园设施，推进保税物流中心（B型）项目建设。加快建设东兴边合区、防城边合区等园区。推动开行至重庆、四川等区域的班列直达线，争取开通至越南沿海港口的直达航线。对接国内国际物流通道，提高便利化通关、便捷化运输等综合性现代物流服务水平。完善多方合作机制。依托东兴试验区、跨境合作区等多个国家级开放平台，扩大与越南等东盟国家的交流合作。推进多式联运“一单制”，推行单证物权化，推动与越南开展“两国一检”试点，办好中越东兴—芒街商贸旅游博览会。

　　构建面向东盟的金融开放主门户。推动面向东盟的跨境金融创新，扩大金融服务业对内对外开放，强化与东盟金融市场和跨境保险合作。完善政银企对接机制，打造良好政银、银企关系。深化“引金入港”战略，综合运用金融手段为实体经济做好服务。谋划建设金融大数据中心、跨境征信平台等跨境金融合作项目。

　　加强区域协同开放合作。巩固“北联行动”成果，深化与西南中南地区交通物流合作，与四川、重庆等省市合作打造飞地园区，建设区域性商贸物流中心和产业合作基地。主动融入粤港澳大湾区建设，高水平承接东部发达地区产业转移。推进海峡两岸产业合作区防城港产业园建设。加快融入北防钦一体化发展，加强与钦州、北海在港口、产业和公共服务等领域的一体化建设，共同打造海边山经济走廊，推动北部湾经济区高质量发展。

　　促进外贸转型升级。落实自治区新一轮“加工贸易倍增计划”，规划布局加工贸易集聚园区，推动边民互市贸易转型升级。建立网上互市区、水果市场，加快发展跨境电商。利用好北仑河二桥、东兴互市贸易区临时浮桥、峒中口岸等新增通关设施，提升边境贸易便利化水平，努力扭转边境口岸运力过剩人多货少的局面。

　　（六）抓好重点领域关键环节改革，增强发展动力活力

　　深化供给侧结构性改革。按照“巩固、增强、提升、畅通”方针，巩固“三去一降一补”成果，增强微观主体活力，提升产业链水平，畅通经济循环。完善市场化、法治化产能退出机制，强化“地条钢”取缔后续监管。落实结构性减税、降低社会保险费率等政策。规范涉企经营服务性收费，力争在水电气降成本方面有新突破。落实增减挂钩、盘活土地等用地政策。加大企业稳岗支持力度，解决好企业用工难问题。

　　深化资源要素市场化配置改革。盘活占而不用、长期闲置或低效的存量资源，优化土地配置，有效增加土地供应量，为项目建设腾挪空间。完成上思耕地占补平衡机制等改革任务。运用基金、债券等各种融资工具，为项目筹集建设资金。解决好市场供需脱节、人力资源与产业需求脱节、金融与实体经济脱节的问题，促进资源要素自由高效流动。

　　深化“放管服”改革。建设招商引资项目代办服务、政企沟通、投诉举报三大平台，力争营商环境进入全区前列。深化商事制度改革，全面实施市场准入负面清单制度，压缩不动产登记时间。进一步完善企业简易注销登记改革试点。深化投资项目审批制度改革，实现项目审批调度和监管“一网服务”。实施政务数据“聚通用”攻坚行动计划，建设数字政务一体化平台，真正让数据多跑路、群众少跑腿、企业好办事。

　　深化国资国企和相关体制改革。按照“调结构、减层级、降负债、强主业”要求，抓好国资国企优化配置。加快四大集团公司整合重组，做好产权划转、工商变更登记等相关工作。抓好“僵尸企业”的处置，及时解决企业改制改组中的关键问题，妥善安置下岗职工。抓好东兴试验区管理体制改革，完成市管开发区体制机制、企沙镇行政管理体制改革任务。扎实推进教育、文化、供销社等其他改革。

　　深化财税金融改革。明晰市县财政权责关系，推进财政事权与支出责任划分改革。深化预算管理改革，提高预算透明度。落实中央税制改革，推进地方税体系建设。用好政府投资引导基金，健全地方金融监管体系。推广“农金村办”模式，助推农村金融改革。推动中越互设跨境金融服务机构，拓展跨境贸易结算业务。

　　深化农村综合改革。完善农村基本经营制度，培育家庭农场和农民合作社等新型农业经营主体。完善农村承包地“三权分置”制度，构建农村产权流转交易市场体系，稳慎推进农村宅基地改革。深化集体经营性资产股份合作制改革，抓好“三变”改革试点。加快农业科技创新，增强农业可持续发展能力。

　　（七）大力发展现代服务业，积极扩大消费需求

　　发展冷链经济拉动消费。以重点供应南北差异化海鲜、水果和肉类等产品为抓手，深挖国内外市场资源和消费需求，拓宽农副产品供应和分销渠道。加密中西部地区冷链专列，恢复拓展我市至越南、泰国等地的水果集装箱航线，形成内外贸冷链集装箱航线2条以上、常态化冷链班列运输路线3条以上、跨境公路冷链运输专线1条以上。规划建设口岸加工物流聚集区，加快建设海产品和水果集散中心，新增冷库库容10万吨以上，打造“一带一路”国际冷链物流枢纽城市。

　　发展旅游拉动消费。加快边境旅游试验区建设，抓好旅游试验区通关便利化、产业发展引导新机制等五大探索工作。充实完善旅游项目库，推动一批文旅重大项目尽快开工建设。继续办好三月三、京族哈节等系列文化旅游活动，培育自驾游、乡村游、康养游等旅游新业态，用好境外旅客离境退税政策，促进旅游留人、留心、留投资，提升旅游消费贡献率。

　　发展边贸加工促进消费。推进边贸转型升级，鼓励农副产品、海产品、红木、中草药等边境贸易企业向贸易加工企业转型，完善“边民互助”合作模式，实现以贸兴工、以工促贸、工贸结合、协调发展，助推边贸消费升级。

　　培育消费新增长点。顺应居民消费提质转型升级新趋势，不断激发潜在消费。激发居民消费潜力。做好养老、家政等消费服务。鼓励和引导农村居民增加交通通信、文化娱乐等消费。改造提升餐饮、商品零售等传统商贸服务业，鼓励新兴消费。合理引导住房消费。发展住房租赁市场，支持机构化住房租赁企业发展，建立政府住房租赁管理服务平台，发展共有产权住房。稳定房地产市场预期，完成棚改任务，多渠道解决群众住房问题。扩大电商消费市场。加快发展智慧商圈、电商社区等新业态，扩大跨境电商业务，推进中国—东盟信息港经贸服务平台建设，抓好电商进农村综合示范县（市、区）创建。

　　（八）统筹协调重点区域开发，发展壮大县域经济

　　推进重点区域开发。优化经开区、高新区与防城工业园区、东兴边合区、上思工业集中区的规划布局建设，经开区、高新区分别统筹开发建设茅岭飞地园区、九龙湖生态科技产业园。统筹推进边境、海港口岸联动发展，串点成线，连线成面，形成集聚效应。统筹协调滨海区域，发掘环东湾、西湾、珍珠湾等优越滨海自然资源，重点做好生态、农业、旅游、近岸海洋等开发建设。利用好十万大山资源优势，推动一二三产业融合发展。

　　强化项目在县域经济中的支撑作用。针对我市县域项目储备少、规模小、落地难的实际，各县（市、区）要把项目工作作为头等大事来抓，围绕重点项目和产业延伸的需求，强化“对外引”招大引强，强化“向上跑”争资争项，强化“全民上”创业创新，努力提高项目数量和质量，增强县域经济的发展动力。

　　统筹协调县域工业发展。根据国家和自治区的要求，每个县（市、区）只设立1个工业园区，可按照一区多园模式建设和管理，原则上下辖不超过3个园区。各县（市、区）要抓住1—3个支柱产业，集中力量搞好一个工业园区。港口、防城和东兴要利用沿海沿边优势，发展临港工业、贸易加工等特色工业。上思要利用好十万大山资源优势，发展碳酸钙、密度板等资源型工业，拉长临港大工业链条。

　　推进县域新型城镇化。按照全面提高城镇化质量的新要求，统筹协调城乡重点区域的开发建设，推动农业转移人口市民化。加快县城中心区和小城镇建设，增强城镇辐射力和要素吸纳力，抓好城镇土地储备，规范土地一级开发运作。

　　（九）强化生态环境保护，全力推动绿色发展

　　坚决打好污染防治攻坚战。打赢蓝天保卫战，加强重点区域大气污染防治，抓好工业废气、港口码头粉尘、建筑施工扬尘、露天烧烤等综合整治。打好碧水保卫战，做好污水与垃圾处理等基础设施建设，提标改造污水处理厂，扩大污水管网覆盖面。抓好“禁养区”范围内规模养殖整治，强化近岸海域综合治理。打好净土保卫战，推进工业企业污染综合治理，有效控制污染土壤的环境风险。引导农民生态种植，减少化肥农药用量。重拳打击洋垃圾及固体废物非法转移倾倒，共同守护我们的美丽家园。

　　实施生态保护和修复。强化海洋生态环境保护和监管，整治东湾、西湾、江山半岛排水口，抓好近岸水域综合治理。扎实做好东湾红树林生态修复等工程。规划建设东湾湿地公园。落实最严格耕地保护制度，加强水土流失综合治理。建设生态环境监测网络。完成全国围填海现状调查的历史遗留问题处理。完成第二次全国污染源普查和第三次国土调查任务。

　　积极发展生态经济。推广循环经济发展模式，推动绿色制造体系建设和固废综合利用，在节能环保、新能源应用和可再生能源开发领域组织实施一批重点项目，发展装配式建筑产业。打好“绿色牌”“长寿牌”，开发绿色、有机高品质农产品，做大“稻田+生态综合种养”和林下经济。倡导生态养殖，推进禽畜养殖废弃物资源化利用。发展环保服务业，加快开放环保治理市场。开展绿色生活行动，持续推进节约型机关建设。

　　强化生态环境保护监管。坚决做好中央环保督察“回头看”和国家海洋督察反馈问题整改，加强生态环境保护督察。深入落实河长制，探索实施湾长制、湖（库）长制。加快生态防城港建设，推动形成人与自然和谐发展新格局，致力建设“城在海中、海在城中、人在景中”美丽中国典范城市，让我们的山更青、水更绿、天更蓝，生态底色永葆亮丽！

　　（十）坚持补短板强弱项，厚植群众获得感幸福感

　　深入实施教育振兴工程。统筹推进义务教育城乡一体化改革发展，加快完善民族贫困地区教育基础设施，年内建成4所义务教育学校。推进高中教育特色化多样化，加快职业教育产教融合。落实乡村教师生活补助标准。建立市教师培训中心，抓好名校长名教师学科带头人的培养，提升教育核心竞争力。

　　打造健康防城港。落实城乡居民基本医保和大病保险制度。深化医药卫生体制改革，持续推进分级诊疗。抓好三级甲等综合医院创建，加快市第一人民医院迁建。加快标准化村卫生室建设，抓好乡村医生定向培养，健全医学人才培训机制。推进国家卫生城市创建。加强健康品牌建设，推动健康产业融合发展，开工建设桂台两岸健康产业城。

　　加强就业和社会保障。对接就业需求，扩大职业技能培训。紧扣双创主题，加强招才引智。支持农民工、退伍军人创业就业。加大城镇困难群众脱困解困力度，做好农民工工资清欠工作。提高困难残疾人补贴和孤儿基本生活最低养育标准。实施全民参保计划，确保应保尽保。

　　繁荣发展文体事业。巩固国家公共文化服务体系示范区创建成果，开展文化惠民活动，创作一批优秀文艺作品。实施乡村文化振兴工程，挖掘乡村非物质文化遗产资源。加强革命文物、历史文化名镇名村及传统村落保护。发展智慧广电，构建数字防城港“广电云”服务体系。继续办好开海节、马拉松、海上龙舟节等节庆活动。

　　认真抓好为民办实事。在抓好去年为民办实事跨年度项目后续建设的同时，今年要扎实做好本次大会表决通过的10个新项目建设。我们要统筹兼顾，克难攻坚，一项项推进，一件件落实，做一事成一事，逐步把基础设施、教育、卫生、文化、交通等领域的历史欠账补齐，让城市的“面子”更靓，“里子”更实，让港城百姓自豪多多、幸福满满！

　　各位代表！边疆的稳定和繁荣责任重大。我们要扎实做好稳边守边固边工作，推进新时代兴边富民行动，发展民族团结进步事业。我们要坚持底线思维，强化风险意识，提高风险化解能力，防范化解经济、社会、外事外经等领域和自然灾害等重大风险。加强应急管理工作，提高安全生产和防灾减灾救灾能力。抓好食品药品安全市场监管。推进平安防城港建设，抓好扫黑除恶、禁毒、反走私等社会治安综合治理。加强精神文明建设，培育和践行社会主义核心价值观，积极创建自治区文明城市。做好双拥工作，认真落实支持退役军人创业税收优惠政策，谋划军民融合发展示范区建设。抓好救灾救济、优抚安置、社会福利和慈善等工作。支持工会、共青团、妇联等群团组织开展工作。做好外事、侨务、决策咨询、统计、人防边海防、气象、档案、地方志等工作。

　　各位代表！新形势新任务对政府工作提出了更高要求，各级政府必须树立“四个意识”，坚定“四个自信”，做到“两个维护”，自觉在思想上政治上行动上同以习近平同志为核心的党中央保持高度一致。加强政府自身建设，提高政府治理能力和治理水平。有序推进市县机构改革，确保今年3月底前基本完成全市机构改革任务。按照自治区部署，扎实开展“重大项目建设攻坚突破年”“产业大招商攻坚突破年”“优化营商环境攻坚突破年”活动。完善“1+4”绩效制度，推动扁平化、极简化、专业化管理，对各项工作实行全区进度排位预警预告，倒逼工作落实和终端见效。自觉接受人大法律和工作监督，支持政协履行政治协商、民主监督、参政议政职能，认真办理人大代表建议和政协提案。推进行政决策科学化民主化法治化。完善容错纠错机制，激励干部敢担当善作为。加强审计监督，规范权力运行。深入贯彻中央“八项规定”及其实施细则精神，力戒形式主义、官僚主义，抓好政府系统廉政建设。进一步精简会议文件，大兴学习调研之风，培养专业能力和专业精神，全面增强“八种本领”，以更强更实的执行力高质量推进各项工作落实。

　　各位代表！风劲扬帆图新志，砥砺奋进正当时。我们已经站在推动改革开放再出发、促进经济高质量发展的新起点上。让我们更加紧密团结在以习近平同志为核心的党中央周围，在自治区党委、政府和市委的坚强领导下，开拓创新，克难攻坚，加快构建海边山现代化经济体系，以优异成绩向中华人民共和国成立70周年献礼！
